# Second-order asymptotics of fractional Gagliardo seminorms as $s\rightarrow 1^-$ and convergence of the associated gradient flows

**DOI:** 10.1007/s13540-025-00472-8

**Published:** 2025-12-03

**Authors:** Andrea Kubin, Valerio Pagliari, Antonio Tribuzio

**Affiliations:** 1 Matematiikan ja Tilastotieteen Laitos, Jyväskylän Yliopisto, Jyväskylä, Finland; 2 Institute of Analysis and Scientific Computing, TU Wien, Wiedner Hauptstraße 8-10, 1040 Vienna, Austria; 3 Institute for Applied Mathematics, University of Bonn, Endenicher Allee 60, 53115 Bonn, Germany

**Keywords:** Gagliardo seminorms, $\Gamma$-convergence, Second-order expansion, Fractional gradient-flows, 49J45, 35R11, 35B40, 45E10, 26A33

## Abstract

We study the second-order asymptotic expansion of the *s*-fractional Gagliardo seminorm as $s\rightarrow 1^-$ in terms of a higher-order nonlocal functional. We prove a Mosco-convergence result for the energy functionals and characterize the domain of the limit, which can be interpreted as the space of functions of differentiability order “logarithmically larger” than one. This is also motivated by the fact that the first variation of the limit is the formal derivative of the (scaled) *s*-fractional Laplacian as $s\rightarrow 1^-$. In the second part of the paper, we show that the $L^2$-gradient flows of the energies converge to the $L^2$-gradient flows of the Mosco-limit.

## Introduction

In recent years, the interest in nonlocal problems has grown significantly in the mathematical community. This is due to the fact that, by their nature, nonlocal energies require less regularity to be well-defined and hence have a broader applicability than their local equivalent. In these terms, *convolution-type* functionals are by now considered as a more general counterpart of elastic-type energies appearing, for instance, in mechanics, micromagnetism and related fields. A challenging question is the study of the asymptotic behaviour of these nonlocal energies as the *interaction horizon* between two points goes to zero.

The prototypical nonlocal energy of convolution-type is the squared *s*-*Gagliardo seminorm*

$$\begin{aligned} [u]_{H^s(\Omega )}^2:=\int _{\Omega }\int _{\Omega } \frac{|u(y) - u(x)|^2}{|y-x|^{N+2s}} \,\textrm{d}y \,\textrm{d}x \end{aligned}$$

where $N\in \mathbb {N}\setminus \{0\}$, $s\in (0,1)$, $u\in L^2(\Omega )$ and $\Omega \subset \mathbb {R}^N$ is an open, bounded, and Lipschitz regular set. This has been proved to converge as $s\rightarrow 1^-$ (after a suitable rescaling) to a multiple of the squared Sobolev seminorm. The first result in this direction is given in the seminal work [6] where it is proved

$$ \lim _{s\rightarrow 1^-}(1-s)[u]_{H^s(\Omega )}^2= \frac{\omega _N}{2} \Vert \nabla u\Vert _{L^2(\Omega )}^2 $$

for every $u\in H^1(\Omega )$ in the sense of pointwise convergence, where $\omega _N$ is the volume of the *N*-dimensional unit ball. Such a result is indeed proved for every exponent $p\in (1,\infty )$ and it is complemented by a treatment of general nonlocal functionals of convolution-type under suitable decay conditions on the kernels. The analogous result in the case of linear growth-conditions, *i.e.* $p=1$, was studied in [22], where it is shown that the pointwise limit is exactly (a multiple of) the total variation norm of $\nabla u$. These results have been proved to hold in the sense of $\Gamma$-convergence in [41] and generalized to the case of arbitrary open sets in [33].

In this paper, we are interested in the analysis of the second order expansion, as $s\rightarrow 1^-$, of the rescaled *s*-Gagliardo seminorm

$$ G_s(u):=(1-s)[u]_{H^s(\mathbb {R}^N)}^2, $$

restricted to functions supported in an open, bounded, Lipschitz set $\Omega \subset \mathbb {R}^N$. To be precise, we prove that the *rate functionals*

$$ \mathscr {G}_s^1(u):= \frac{G_1(u)-G_s(u)}{1-s}, \quad \text {where } G_1(u):=\frac{\omega _N}{2}\Vert \nabla u\Vert _{L^2(\mathbb {R}^N)}^2, $$

$\Gamma$-converge as $s\rightarrow 1^-$ with respect to the strong $L^2$-topology to the nonlocal limit energy

$$ \mathscr {G}^1(u) := \int _{\mathbb {R}^N}\int _{\mathbb {R}^N}\frac{|\nabla u(x)\cdot h|^2\chi _{B(0,1)}(h)-|u(x+h)-u(x)|^2}{|h|^{N+2}}\,\textrm{d}h\,\textrm{d}x. $$

Our limit result (cf. Theorem 2), supported by the $L^2$-equicoercivity of the $\mathscr {G}_s^1$ energies, upgrades to a *Mosco-limit*. We complement this analysis by characterizing the domain of $\mathscr {G}^1$ (cf. Lemma 2), exploiting its Fourier representation, which consists of the functions $u\in H^1_0(\Omega )$ whose Fourier transform ${\hat{u}}$ complies with

$$ \int _{\mathbb {R}^N} |\xi |^2\log (1+|\xi |^2)|{\hat{u}}(\xi )|^2\,\textrm{d}\xi <+\infty . $$

Our results conclude the asymptotic analysis of the *s*-Gagliardo seminorm up to the second order in the singular limit $s\rightarrow 1^-$. To the best of our knowledge, our limit result introduces the *new* quadratic, nonlocal energy $\mathscr {G}^1$, which is close to other $L^1$-based analogs appearing in the second-order study of nonlocal perimeters [14, 30]. A novel contribution of our work (which is yet missing in the linear-growth case) is the precise characterization of the domain of the limit energy. As it is apparent from this characterization, we can interpret $\mathscr {G}^1$ as an $L^2$-based energy of differentiability order “logarithmically larger” than one. This can also be formally explained by testing out the energy on Hölder regular functions since the singularity of both terms in $\mathscr {G}^1$ (as $|h|\rightarrow 0$) cancels in the difference if *u* is sufficiently differentiable. Indeed, taking $u\in C^{1,\alpha }_c(\Omega )$, by Taylor’s expansion and the Fundamental Theorem of Calculus, it holds that $u(x+h)=u(x)+\nabla u(x)\cdot h + O(|h|^{1+\alpha })$ hence

$$\begin{aligned} \mathscr {G}^1(u)= &   \int _{\mathbb {R}^N}\int _{B(0,1)}\frac{(\nabla u(x)\cdot h)O(|h|^{1+\alpha })}{|h|^{N+2}} \,\textrm{d}h\,\textrm{d}x \\  &   -\int _{\mathbb {R}^N}\int _{B(0,1)^c}\frac{|u(x+h)-u(x)|^2}{|h|^{N+2}}\,\textrm{d}h\,\textrm{d}x, \end{aligned}$$

which is finite for any $\alpha >0$.

An alternative interpretation of the functional $\mathscr {G}^1_s$, which further justify the interest in its asymptotics, is that of an interaction energy consisting of two competing contributions; a local attractive-term and a nonlocal repulsive one. For this reason, the functional $\mathscr {G}^1_s$ is closely related to the energies arising in the study of liquid drop models (see [17] for an introduction).

We conclude the discussion on our main $\Gamma$-convergence result by briefly commenting on possible variants of this work. One first question is whether our analysis could be carried over also to non-quadratic functionals of *p*-growth with $p\in (1,\infty )$, $p\ne 2$, as also in this case first-order $\Gamma$-convergence results are present [9, 41]. We expect the analogous analysis to hold true, with essentially the same proof strategy. However, in this case, we lack the tool of the Fourier transform, thus the characterization of the domain would require a different approach (*e.g.* via Besov or Triebel-Lizorkin spaces, see [10]). We therefore postpone this to future works.

The second step in our analysis is the study of the $L^2$-gradient flows associated to $\mathscr {G}_s^1$. As one may expect from the static asymptotic analysis, the *parabolic-type* flows governed by the “gradient” of $\mathscr {G}_s^1$ converge as $s\rightarrow 1^-$ to the gradient flow of $\mathscr {G}^1$ (see Theorem 4). In this context, thanks to the $\lambda$-*convexity* of the functionals involved, the correct notion of gradient of the energies $\mathscr {G}_s^1$ and $\mathscr {G}^1$ is that of *first variation*. For regular test functions $\phi \in C^\infty _c(\Omega )$, the first variation of $\mathscr {G}_s^1$ is

$$\begin{aligned} \frac{ d }{d t} \mathscr {G}_s^1(u+t\phi )|_{t=0}=\omega _N\Big \langle u,\frac{(-\Delta )-\sigma _{N,s}(-\Delta )^s}{1-s}\phi \Big \rangle , \end{aligned}$$

where $(-\Delta )^s$ is the *s*-fractional Laplace operator and $\sigma _{N,s}>0$ is a scaling factor converging to 1 as $s\rightarrow 1^-$. At least formally, this converges to the first variation of the $\Gamma$-limit $\mathscr {G}^1$ which introduces a *new* operator defined by

$$\begin{aligned} \mathfrak {L} \phi (x):= &   -2N \omega _N \phi (x)+ 4\int _{B(0,1)^c} \frac{\phi (x+h)}{\vert h \vert ^{N+2}}\,\textrm{d}h \\  &   - 2\int _{B(0,1)} \frac{2\phi (x)-\phi (x-h)-\phi (x+h)+ h \cdot \nabla ^2 \phi (x)h }{\vert h \vert ^{N+2}} \,\textrm{d}h, \end{aligned}$$

for every $\phi \in C^\infty _c(\Omega )$. This operator can be seen as the *formal derivative* as $s\rightarrow 1^-$ of $\sigma _{N,s}(-\Delta )^s$ and can be considered as a counterpart to the *logarithmic Laplacian* $\log (-\Delta )$ [16] obtained as a formal derivative of $(-\Delta )^s$ as $s\rightarrow 0^+$ (see also [19] for a variational point of view in this case). We discuss this interpretation (and possible outlooks) in Remark 2 below. In this work, we treat this limit of operators as a stability result of the flows, obtained as an application of the approach (from general Hilbert spaces) developed in [19], where it is proved that uniform convexity assumptions (*i.e.* $\lambda$-convexity and $\lambda$-positivity) are sufficient conditions to guarantee that $\Gamma$-convergence commutes with the gradient-flow structure, exploiting the minimizing-movements and energy dissipation approaches [3, 18, 42].

The literature on local limits of fractional-type functionals, in both the critical limits $s\rightarrow 0$ and $s\rightarrow 1$, is by now very vast. Among the many results, we mention the pivotal papers [2, 41] in which the asymptotic behavior of (relative) *fractional perimeters* as $s\rightarrow 1^-$ is studied in terms of $\Gamma$-convergence (see also [26] for analogous results in the supercritical case $s\rightarrow 1^+$ and [31] in which sets with fractional perimeters are characterized via fractional heat flows). In this context, also results on the second order asympotics can be found in [14] for the classical fractional perimeter and in [30] for more general convolution energies related to the study of thin ferromagnetic films (see also [15] for energies associated with $L^1$ kernels with finite second moment). In this direction, regularity properties of certain local minimizers of the fractional perimeter have been investigated in [12]. The first result regarding the limit as $s \rightarrow 0^+$ is [36], in which the authors show that the squared *s*-Gagliardo seminorm (rescaled by *s*) pointwise converge to (a multiple of) the squared $L^2$-norm (see also [13, 25] for similar results in the context of *s*-fractional perimeters). This asymptotic analysis has been developed also in terms of $\Gamma$-convergence in [19] for the standard *s*-Gagliardo seminorm and in [28] for fractional perimeters both in the first and in the second order. In this latter case, these studies provided two new nonlocal functionals, referred to as 0-*fractional perimeter* in [28] and its corresponding $L^2$-based version in [19, Theorem 1.4]. Many variants of the problem have also been considered, for instance incorporating anisotropies [34], or having different interaction potentials [4, 5, 27, 35]. For other related nonlocal-to-local results see also [1, 20, 23, 37] and the references therein.

The paper is structured as follows: after recalling some known results in the literature in Section 2, in Section 3 we study the Mosco-convergence of the functionals $\mathscr {G}^1_s$ as $s\rightarrow 1^-$ to $\mathscr {G}^1$. Then, in Section 4, we prove the convergence of the gradient flows of $\mathscr {G}_s^1$ to the gradient flows of $\mathscr {G}^1$.

**Notation of the paper:** For $N\in \mathbb {N}\setminus \{0\}$, our analysis is settled in the *N*-dimensional space $\mathbb {R}^N$. We denote the Euclidean inner product between $a,b\in \mathbb {R}^N$ as $a\cdot b$ and the corresponding norm as |*a*|. We denote by *B*(*x*, *r*) the open ball in $\mathbb {R}^N$ of center *x* and radius *r*. We write $B(x,r)^c$ for the complement of *B*(*x*, *r*), while for the topological boundary of *B*(0, 1) we use the symbol $\mathbb {S}^{N-1}$ or $\partial B(0,1)$. We denote by $\mathscr {L}^N$ the *N*-dimensional Lebesgue measure, and for $k \in \{0,\dots , N-1\}$ we denote ${\mathcal {H}}^k$ the *k*-dimensional Hausdorff measure. We set $\omega _N:=\mathscr {L}^N(B(0,1))$, so that the $(N-1)$-dimensional Hausdorff measure of $\mathbb {S}^{N-1}$ equals $N\omega _N$. Throughout the paper, we write $C(*,\ldots ,*)$ to indicate a generic positive constant that depends only on $*,\ldots ,*$ and that may change from line to line.

## The subcritical rate of convergence of the Gagliardo seminorms

In this section we analyze the asymptotic behaviour of the scaled *s*-Gagliardo seminorms as $s\rightarrow 1^-$. Let $s\in (0,1)$, we define the scaled *s*-Gagliardo seminorm of a measurable function $u : \mathbb {R}^N \rightarrow \mathbb {R}$ as

2.1 $$\begin{aligned} G_s(u):=(1-s) \int _{\mathbb {R}^N} \int _{\mathbb {R}^N} \frac{| u(y)-u(x) |^2}{| x-y |^{N+2s}}\,\textrm{d}y \,\textrm{d}x. \end{aligned}$$

For simplicity, we will consider compactly supported functions but the same analysis can be performed by using local convergences.

We fix $\Omega$ an open bounded subset of $\mathbb {R}^N$ with Lipschitz continuous boundary as our ambient space. In what follows, for every $v\in L^2(\Omega )$ we denote without relabeling the $L^2(\mathbb {R}^N)$ function which is extended to zero outside $\Omega$.

### Preliminary (known) results and first-order limits

The asymptotic analysis of the family $\{G_{s_n}\}_{n \in \mathbb {N}}$ as $s_n\rightarrow 0^+$ and as $s_n\rightarrow 1^-$ in the sense of $\Gamma$-convergence has already been carried out in the literature. In this respect, we recall the following results.

#### Theorem 1

Let $\{s_n\}_{n \in \mathbb {N}}$ be a sequence such that $s_n \rightarrow 1^-$ as $n \rightarrow + \infty$. Then, the following hold.

1. (compactness) Let $\{u_n\}_{n \in \mathbb {N}}\subset L^2(\Omega )$ satisfy
  $$ \Vert u_n \Vert _{L^2(\Omega )} + G_{s_n}(u_n) \le M $$
  for some $M\ge 0$ independent of *n*. Then, there exists $u \in H_{0}^1(\Omega )$ such that, up to subsequences, $u_n \rightarrow u$ strongly in $L^2(\Omega )$.
2. ($\Gamma$-convergence) The sequence $\{G_{s_n}\}_{n \in \mathbb {N}}$ $\Gamma$-converges with respect to the strong $L^2(\Omega )$-topology to the functional $G_1$ defined as
  2.2 $$\begin{aligned} G_1(u):={\left\{ \begin{array}{ll} \displaystyle { \frac{\omega _N}{2} \int _{\Omega } \vert \nabla u(x) \vert ^2 \,\textrm{d}x} &  \text {if } u \in H_0^1(\Omega ) \, , \\ +\infty &  \text {otherwise in } L^2(\Omega ). \end{array}\right. } \end{aligned}$$

For similar compactness and $\Gamma$-convergence results, for general convolution-type energies, see also [41]. An analogous $\Gamma$-convergence result in the setting of the fractional perimeter has been studied in [2]. For the analogous result corresponding to the singular limit $s\rightarrow 0^+$ we refer the interested reader to [19, Theorem 1.2].

For $u \in L^2(\Omega )$, we introduce the rate functionals $\mathscr {G}^1_s: L^2(\Omega )\rightarrow (-\infty ,+\infty ]$ defined as

2.3 $$\begin{aligned} \mathscr {G}^1_s(u) :={\left\{ \begin{array}{ll} \displaystyle \frac{G_1(u)-G_s(u)}{1-s} &  \text {if } u\in H^1_0(\Omega ) \\ +\infty &  \text {otherwise in } L^2(\Omega ). \end{array}\right. } \end{aligned}$$

The second order asymptotics of $G_s$ as $s\rightarrow 0^+$ has been already established in the literature in terms of $\Gamma$-convergence (see [19, Theorem 1.4] and [28] for the case of fractional perimeters). In the sequel, we will then focus on the analysis of $\{\mathscr {G}_{s}^1\}_{s\in (0,1)}$ as $s\rightarrow 1^-$.

## The main limit result

We will prove that the energies $\mathscr {G}_s^1$ *Mosco-converge* to a limit functional $\mathscr {G}^1$. We remind that Mosco-convergence (see [38]), *i.e.* when weak $\Gamma$-liminf and strong $\Gamma$-limsup coincide, implies $\Gamma$-convergence.

Before stating the main convergence result, we first define and discuss the limit functional $\mathscr {G}^1$, since its formulation is quite involved.

### The candidate Mosco-limit

We introduce the candidate $\Gamma$-limit $\mathscr {G}^1: L^2(\Omega ) \rightarrow (-\infty , \, + \infty ]$ defined as

3.1 $$\begin{aligned} \begin{aligned} \mathscr {G}^1(u)&:= \int _{\mathbb {R}^N}\int _{B(0,1)}\frac{\int _0^1|\nabla u(x+th)\cdot h|^2\,\textrm{d}t-|u(x+h)-u(x)|^2}{|h|^{N+2}}\,\textrm{d}h\,\textrm{d}x \\&\qquad - \int _{\mathbb {R}^N}\int _{B(0,1)^c}\frac{|u(x+h)-u(x)|^2}{|h|^{N+2}}\,\textrm{d}h\,\textrm{d}x, \end{aligned} \end{aligned}$$

if $u \in \mathfrak {D}(\mathscr {G}^1) \subset L^2(\Omega )$ and $\mathscr {G}^1(u)=+\infty$ if $u \in L^2(\Omega ) \setminus \mathfrak {D}(\mathscr {G}^1)$, where $\mathfrak {D}(\mathscr {G}^1)$ is the set of functions $u \in H_0^1(\Omega )$ such that

3.2 $$\begin{aligned} \frac{ \int _{0}^{1} \vert \nabla u(x+th) \cdot h \vert ^2 \,\textrm{d}t-\vert u(x+h)-u(x)\vert ^2}{\vert h \vert ^{N+2}} \chi _{B(0,1)}(h) \in L^1(\mathbb {R}^N \times \mathbb {R}^N). \end{aligned}$$

#### Remark 1

The limit functional $\mathscr {G}^1$, as defined in (3.3), can be written (inside $\mathfrak {D}(\mathscr {G}^1)$) in an equivalent form as

3.3 $$\begin{aligned} \begin{aligned} \mathscr {G}^1(u) = \int _{\mathbb {R}^N}\int _{\mathbb {R}^N}\frac{|\nabla u(x)\cdot h|^2\chi _{B(0,1)}(h)-|u(x+h)-u(x)|^2}{|h|^{N+2}}\,\textrm{d}h\,\textrm{d}x, \end{aligned} \end{aligned}$$

by exploiting Fubini’s Theorem and a change of variable in the gradient term. Indeed, by (3.2) we can switch the orders of the integrals and using the change of variable $x'=x+th$ we get

$$ \int _{R^N}\int _{B(0,1)}\int _0^1\frac{|\nabla u(x+th)\cdot h|^2}{|h|^{N+2}}\,\textrm{d}t\,\textrm{d}h\,\textrm{d}x = \int _{R^N}\int _{B(0,1)}\frac{|\nabla u(x')\cdot h|^2}{|h|^{N+2}}\,\textrm{d}h\,\textrm{d}x'. $$

Although identity (3.3) is more compact, the main advantage of the expression in (3.1) is the fact that the function in (3.2) is non-negative by a straightforward application of the Fundamental Theorem of Calculus and Jensen’s inequality. Also, in (3.1) we separate the singular integral (first term) from the absolutely convergent one (second term).

For these reasons, we prefer formulation (3.1) in the sequel, especially in computations.

Even though the characterization of the domain $\mathfrak {D}(\mathscr {G}^1)$ is quite involved, we can prove that this set is dense in $L^2(\Omega )$ thanks to the following result.

#### Lemma 1

Let $$u \in C_c^{2}(\Omega )$$. Then

$$ \frac{ \int _{0}^{1} \vert \nabla u(x+th) \cdot h \vert ^2 \,\textrm{d}t-\vert u(x+h)-u(x)\vert ^2}{\vert h \vert ^{N+2}} \chi _{B(0,1)}(h) \in L^{1}(\mathbb {R}^N \times \mathbb {R}^N), $$

i.e. $$C_c^2(\Omega ) \subset \mathfrak {D}(\mathscr {G}^1)$$.

#### Proof

We preliminarily observe that for every $h\in B(0,1)$

3.4 $$\begin{aligned} \begin{aligned}&\frac{\int _{0}^{1} \vert \nabla u(x_{t,h}) \cdot h \vert ^2 \,\textrm{d}t-\vert u(x_h)-u(x)\vert ^2}{\vert h \vert ^{N+2}} \\&\quad =\int _{0}^1 \frac{|u(x_h)-u(x)- \nabla u(x_{t,h})\cdot h| |u(x_h)-u(x)+\nabla u(x_{t,h})\cdot h|}{\vert h \vert ^{N+2}} \,\textrm{d}t, \end{aligned} \end{aligned}$$

where we also used that the left-hand side is nonnegative. Here and in the rest of the proof we use the notation $x_h=x+h$ and $x_{t,h}=x+th$ to shorten the formulas. By the regularity of *u*, we have that for all $t\in [0,1]$

3.5 $$\begin{aligned} \vert u(x_h)-u(x)-\nabla u(x_{t,h})\cdot h \vert \le R \vert h \vert ^2 \end{aligned}$$

where

$$ R:= C(N)\sup \Big \{ \Big \vert \frac{\partial ^2 u}{\partial x_i \, \partial x_j}(x) \Big \vert \, : x \in \Omega , \, i,j = 1, \dots ,N \Big \}. $$

An application of the triangle inequality on the right-hand side of (3.4) yields

3.6 $$\begin{aligned} \begin{aligned}&\int _{\mathbb {R}^N}\int _{B(0,1)}\frac{\int _{0}^{1} \vert \nabla u(x_{t,h}) \cdot h \vert ^2 \,\textrm{d}t-\vert u(x_h)-u(x)\vert ^2}{\vert h \vert ^{N+2}} \,\textrm{d}h \,\textrm{d}x \\&\quad \le \int _{\mathbb {R}^N} \int _{B(0,1)} \int _{0}^1\frac{\vert u(x_h)-u(x)-\nabla u(x_{t,h})\cdot h \vert \vert u(x_h)-u(x) \vert }{\vert h \vert ^{N+2}} \,\textrm{d}t \,\textrm{d}h \,\textrm{d}x \\&\qquad + \int _{\mathbb {R}^N} \int _{B(0,1)} \int _{0}^1\frac{\vert u(x_h)-u(x)-\nabla u(x_{t,h})\cdot h \vert \vert \nabla u(x_{t,h}) \cdot h \vert }{\vert h \vert ^{N+2}} \,\textrm{d}t \,\textrm{d}h \,\textrm{d}x. \end{aligned} \end{aligned}$$

It is now sufficient to control the two terms at the right-hand side of (3.6). Regarding the first, from (3.5) we infer

$$\begin{aligned} \begin{aligned} \int _{\mathbb {R}^N}&\int _{B(0,1)}\int _{0}^1\frac{\vert u(x_h)-u(x)-\nabla u(x_{t,h})\cdot h \vert \vert u(x_h)-u(x) \vert }{\vert h \vert ^{N+2}} \,\textrm{d}t \,\textrm{d}h \,\textrm{d}x\\&\le R \int _{\mathbb {R}^N}\int _{B(0,1)}\frac{\vert u (x_h)-u(x) \vert }{\vert h \vert ^{N}} \,\textrm{d}h\,\textrm{d}x \\&\le R \int _{\mathbb {R}^N} \int _{B(0,1)} \int _{0}^{1} \frac{\vert \nabla u(x_{s,h})\cdot h \vert }{\vert h \vert ^{N}} \,\textrm{d}s \,\textrm{d}h \,\textrm{d}x\\&\le R \int _{\mathbb {R}^N} \vert \nabla u (\xi ) \vert \,\textrm{d}\xi \int _{B(0,1)} \frac{1}{\vert h \vert ^{N-1}} \,\textrm{d}h= R N \omega _N \int _{\mathbb {R}^N} \vert \nabla u (\xi ) \vert \,\textrm{d}\xi . \end{aligned} \end{aligned}$$

Arguing similarly as above we also get

$$\begin{aligned} \begin{aligned} \int _{\mathbb {R}^N}&\int _{B(0,1)} \int _{0}^1\frac{\vert u(x_h)-u(x)-\nabla u(x_{t,h})\cdot h \vert \vert \nabla u(x_{t,h}) h \vert }{\vert h \vert ^{N+2}} \,\textrm{d}h \,\textrm{d}t \,\textrm{d}x\\&\le R \int _{\mathbb {R}^N} \int _{B(0,1)}\frac{\vert \nabla u (\xi ) \vert }{\vert h \vert ^{N-1}} dh \,\textrm{d}\xi = R N \omega _N \int _{\mathbb {R}^N} \vert \nabla u (\xi ) \vert \,\textrm{d}\xi . \end{aligned} \end{aligned}$$

Thus, by the two formulas above and (3.6) we obtain the claim. $\square$

### Characterization of the domain via Fourier transform

As we already commented on, the definition of $\mathfrak {D}(\mathscr {G}^1)$ is not very transparent, even though, as Lemma 1 shows, it is well-defined in $L^2(\Omega )$, in particular it is dense in the strong $L^2$-topology. The definition of the set $\mathfrak {D}(\mathscr {G}^1)$ can be clarified by making use of the Fourier transform.

Nonlocal, $L^2$-based quadratic functionals can be rewritten in a local form thanks to the Fourier transform see [24, 29]. To fix notation, for every $u\in C^\infty _c(\mathbb {R}^N)$ we define its Fourier transform as

$$ {\mathcal {F}}u(\xi ):=(2\pi )^{-\frac{N}{2}}\int _{\mathbb {R}^N} e^{-i x\cdot \xi } u(x) dx, $$

and this extends (as a linear operator) to the whole $L^2(\mathbb {R}^N)$. When there is no risk of confusion we will write ${\hat{u}}(\xi )={\mathcal {F}}u(\xi )$.

Noticing that ${\mathcal {F}}(u(\cdot +h)-u)(\xi )=(e^{-i h\cdot \xi }-1){\hat{u}}(\xi )$ and that ${\mathcal {F}}(\nabla u)(\xi )=i\xi {\hat{u}}(\xi )$, the Fourier transform (in *x* for fixed *h* and *t*) of the function in (3.2) reads $|h|^{-N-2}\big (|\xi \cdot h|^2-2+2\cos (\xi \cdot h)\big )|{\hat{u}}(\xi )|^2$. Denoting as

3.7 $$\begin{aligned} m(\xi ):=\int _{B(0,1)}\frac{|\xi \cdot h|^2-2+2\cos (\xi \cdot h)}{|h|^{N+2}}\,\textrm{d}h, \end{aligned}$$

by Plancherel’s and Fubini’s Theorems we get that for every $u\in H^1_0(\Omega )$, $u\in \mathfrak {D}(\mathscr {G}^1)$ if and only if

3.8 $$\begin{aligned} \int _{\mathbb {R}^N} m(\xi )|{\hat{u}}(\xi )|^2 \,\textrm{d}\xi <+\infty . \end{aligned}$$

By the change of variable $\eta =|\xi |h$, we can rewrite *m* as

3.9 $$\begin{aligned} m(\xi )=|\xi |^2\int _{B(0,|\xi |)}\frac{|\bar{\xi }\cdot \eta |^2-2+2\cos (\bar{\xi }\cdot \eta )}{|\eta |^{N+2}}\,\textrm{d}\eta \end{aligned}$$

where $\bar{\xi }= \frac{ \xi }{\vert \xi \vert }$. From this we can see that (3.8) is a differentiability condition of order strictly higher than one. By a careful analysis of the decay of $m(\xi )$, we can give a complete characterization of the domain $\mathfrak {D}(\mathscr {G}^1)$.

#### Lemma 2

Let $u\in H^1_0(\Omega )$. Then (3.2) holds if and only if

3.10 $$\begin{aligned} \int _{\mathbb {R}^N}|\xi |^2\log (|\xi |^2+1)|{\hat{u}}(\xi )|^2 \,\textrm{d}\xi <\infty . \end{aligned}$$

In particular $\mathfrak {D}(\mathscr {G}^1)=\{u\in H^1_0(\Omega ) : (3.10) \text { holds true}\}$ and $(\mathfrak {D}(\mathscr {G}^1), \Vert \cdot \Vert _{\mathfrak {D}(\mathscr {G}^1)})$ with $\Vert u \Vert _{\mathfrak {D}(\mathscr {G}^1)} := \Vert \nabla u \Vert _{L^2(\Omega )}+ (\int _{\mathbb {R}^N}|\xi |^2\log (|\xi |^2+1)|{\hat{u}}(\xi )|^2 \,\textrm{d}\xi )^{\frac{1}{2}}$ is a Banach space.

#### Proof

The result follows by providing upper and lower bounds for the multiplier defined in (3.9).

We start by giving an upper bound. Consider first the case $|\xi |\le 2$. Then, since $\cos (t)\le 1-\frac{t^2}{2}+\frac{t^4}{24}$ for every $t\in \mathbb {R}$, we get

$$\begin{aligned} m(\xi ) \le |\xi |^2 \frac{1}{12}\int _{B(0,|\xi |)}\frac{|\bar{\xi }\cdot \eta |^4}{|\eta |^{N+2}}\,\textrm{d}\eta \le \frac{N\omega _N}{24}|\xi |^4. \end{aligned}$$

For $|\xi |>2$ we thus have

$$\begin{aligned} m(\xi )&\le \frac{1}{6}N\omega _N|\xi |^2 + |\xi |^2\int _{B(0,|\xi |)\setminus B(0,2)}\frac{|\bar{\xi }\cdot \eta |^2}{|\eta |^{N+2}}\,\textrm{d}\eta \\&\le \frac{1}{6}N\omega _N|\xi |^2 + N\omega _N |\xi |^2\int _1^{|\xi |}\frac{1}{t}\,\textrm{d}t = N\omega _N|\xi |^2 \Big ( \frac{1}{6}+\log (|\xi |)\Big ). \end{aligned}$$

Hence for some constant $C_2$ depending on *N* it holds

$$ m(\xi ) \le C_2|\xi |^2\big (1+\log (|\xi |^2+1)\big ). $$

We now pass to the lower bound. We preliminarily notice that, straightforward computations yield that $t^2-2+2\cos (t)\ge \frac{5}{9}t^2$, for every $t\in \mathbb {R}$ with $|t|\ge 3$, while for $|t|< 3$ we have $t^2-2+2\cos (t)\ge \frac{1}{48}t^4$. We also denote $E_\xi :=\{\eta \in \mathbb {R}^N\setminus \{0\}:\sqrt{2}\bar{\xi }\cdot \eta \ge |\eta |\}$. Hence, for $|\xi |<3$ we have

$$\begin{aligned} m(\xi )&\ge |\xi |^2 \frac{1}{48} \int _{B(0,|\xi |)}\frac{|\bar{\xi }\cdot \eta |^4}{|\eta |^{N+2}}\,\textrm{d}\eta \\&\ge |\xi |^2 \frac{1}{192}\int _{B(0,|\xi |)\cap E_\xi }\frac{1}{|\eta |^{N-2}}\,\textrm{d}\eta \ge \frac{N\omega _N}{768}|\xi |^4. \end{aligned}$$

If instead $|\xi |\ge 3$ we get

$$\begin{aligned} m(\xi )&\ge \frac{1}{768}N\omega _N|\xi |^4+|\xi |^2 \frac{5}{9} \int _{B(0,|\xi |)\setminus B(0,3)}\frac{|\bar{\xi }\cdot \eta |^2}{|\eta |^{N+2}}\,\textrm{d}\eta \\&\ge \frac{1}{768}N\omega _N|\xi |^4+|\xi |^2 \frac{5}{18}\int _{\big (B(0,|\xi |)\setminus B(0,3)\big )\cap E_\xi }\frac{1}{|\eta |^{N}}\,\textrm{d}\eta \\&\ge \frac{1}{768}N\omega _N|\xi |^4+\frac{5}{36}N\omega _N|\xi |^2(\log (|\xi |)-\log (3)). \end{aligned}$$

This implies that there exists a constant $C_1$ depending on *N* such that

$$ m(\xi ) \ge C_1 |\xi |^2 \big (\log (|\xi |^2+1)-1\big ) $$

and the result is proven. $\square$

### Second-order $\Gamma$-convergence of Gagliardo seminorms

We are now in position to state our result, that is the second-order asymptotics, via $\Gamma$-convergence, of the *s*-Gagliardo seminorms. As said before, we will prove a stronger result, namely the Mosco-convergence of the functionals $\mathscr {G}^1_s$ as $s\rightarrow 1^-$.

#### Theorem 2

Let $\{s_n\}_{n \in \mathbb {N}} \subset (0,1)$ be such that $s_n \rightarrow 1^-$ as $n \rightarrow + \infty$, and let $\mathscr {G}^1_s$ and $\mathscr {G}^1$ be defined as in (2.3) and (3.1) respectively. Then, the following Mosco-convergence results hold true.

1. (compactness) Let $\{u_n\}_{n\in \mathbb {N}}\subset L^2(\Omega )$ be such that
  $$\begin{aligned} \sup _{n \in \mathbb {N}} \mathscr {G}^1_{s_n}(u_n)<+\infty \quad \text {and} \quad \sup _{n \in \mathbb {N}} \Vert u_n\Vert _{L^2(\Omega )}<+\infty . \end{aligned}$$
  Then, up to subsequences, $u_n \rightharpoonup u$ in the weak topology of $H^1_0(\Omega )$ for some $u\in \mathfrak {D}(\mathscr {G}^1)$.
2. (weak liminf inequality) For every $u \in L^2(\Omega )$ and for every $\{u_n\}_{n \in \mathbb {N}} \subset L^2(\Omega )$ with $u_n \rightharpoonup u$ in the weak topology of $L^2(\Omega )$, it holds
  $$\begin{aligned} \mathscr {G}^1(u)\le \liminf _{n \rightarrow + \infty } \mathscr {G}^1_{s_n}(u_n). \end{aligned}$$
3. (strong limsup inequality) For every $u \in L^2(\Omega )$ there exists $\{u_n\}_{n \in \mathbb {N}} \subset L^2(\Omega )$ with $u_n \rightarrow u$ in $L^2(\Omega )$ such that
  $$\begin{aligned} \mathscr {G}^1(u)\ge \limsup _{n \rightarrow + \infty } \mathscr {G}^1_{s_n}(u_n). \end{aligned}$$

The next subsections are devoted to the proof of Theorem 2.

An analogous result in the case of $\Gamma$-convergence of fractional perimeters has been obtained in [14]. Our strategy adapts the one in that work to the current setting.

#### Coercivity and lower semicontinuity of $\mathscr {G}_s^1$

In the next proposition we prove the lower semicontinuity of the functionals $\mathscr {G}_s^1$ for fixed $s\in (0,1)$. We also provide a compactness result (in the weak $H^1$-topology) for sequences with bounded $L^2$-norm and bounded $\mathscr {G}_s^1$-energy.

##### Proposition 1

Let $s \in (0,1)$ be fixed and let $\mathscr {G}^1_s$ be defined as in (2.3). Let $\{u_n\}_{n \in \mathbb {N}}\subset L^2(\Omega )$ be such that

3.11 $$\begin{aligned} \Vert u_n \Vert _{L^2(\Omega )} \le m \quad \text {and} \quad \mathscr {G}^1_s(u_n) \le M \end{aligned}$$

for some *M*, $m>0$ and for every $n \in \mathbb {N}$, then $\Vert \nabla u_n\Vert _{L^2(\Omega )} \le M'$ for every $n \in \mathbb {N}$, for some positive constant $M'$ depending on *M*, *s* and *N*.

In particular, the sequence $\{u_n\}_{n \in \mathbb {N}}$ converges in the weak $H^1$-topology, up to a subsequence, to a function $u\in H^1_0(\Omega )$.

Moreover, the functional $\mathscr {G}_s^1$ is lower semicontinous with respect to the weak $L^2$-topology.

##### Proof

Notice first that for every $u \in H^1_0(\Omega )$ with $\Vert u \Vert _{L^2(\Omega )} \le m$ by the interpolation inequality, see [32], we have

3.12 $$\begin{aligned} \begin{aligned} \int _{\mathbb {R}^N} \int _{\mathbb {R}^N} \frac{\vert u(x)-u(y)\vert ^2}{\vert x-y \vert ^{N+2s}}\, \,\textrm{d}x \,\textrm{d}y&\le C\Vert u \Vert _{L^2(\Omega )}^{2(1-s)} \Vert \nabla u \Vert _{L^2(\Omega )}^{2 s} \\&\le Cm^{2(1-s)} \Vert \nabla u \Vert _{L^2(\Omega )}^{2 s} \\&=Cm^{2(1-s)}N^s \big (G_1(u)\big )^{s}. \end{aligned} \end{aligned}$$

Let $\{u_n\}_{n\in \mathbb {N}}$ be as in (3.11), by definition of $\mathscr {G}_s^1$ and by (3.12) we have

$$\begin{aligned} (1-s)M \ge G_1(u_n)-G_s(u_n) \ge G_1(u_n)-(1-s)Cm^{2(1-s)}N^s \big (G_1(u_n)\big )^{s}. \end{aligned}$$

From this we conclude that necessarily $\Vert \nabla u_n\Vert _{L^2(\Omega )} = \big (\frac{2}{\omega _N}G_1(u_n)\big )^\frac{1}{2} \le M'$, where $M'$ is a constant which depends on *m*, *M*, *s* and *N*. As a consequence, by the Rellich-Kondrachov theorem, we have that $u_n$ is compact in the weak $H^1$-topology and its cluster points are in $H^1_0(\Omega )$ by (3.11).

Now, consider a sequence $\{v_n\}_{n\in \mathbb {N}}\subset L^2(\Omega )$ such that $v_n \rightharpoonup v$ in $L^2(\Omega )$ and that $\mathscr {G}_s^1(v_n) \le M$. Since, in particular $\Vert v_n\Vert _{L^2(\Omega )}$ is bounded, by the previous argument we get that $\Vert \nabla v_n\Vert _{L^2(\Omega )} \le M'$ where $M'$ depending on *M*, *s*, and *N*. Again by the Rellich-Kondrachov theorem we have and that $v_n \rightarrow u$ in $L^2(\Omega )$. Notice now that, by Hölder’s inequality, we get

$$\begin{aligned}&|G_s(v_n)-G_s(v)| \\&\quad \le \int _{\mathbb {R}^N}\int _{\mathbb {R}^N}|(v_n(x)+v(x))-(v_n(y)+v(y))| \\&\quad \qquad \qquad \qquad \times |(v_n(x)-v(x))-(v_n(y)-v(y))||x-y|^{-N-2s}\,\textrm{d}x \,\textrm{d}y \\&\quad \le \big (G_s(v_n+v)\big )^\frac{1}{2} \big (G_s(v_n-v)\big )^\frac{1}{2}. \end{aligned}$$

Using again the interpolation inequality as in the first line of (3.12) we obtain

$$ |G_s(v_n)-G_s(v)| \le {\tilde{C}} \Vert v-v_n\Vert _{L^2(\Omega )}^{2(1-s)} $$

for some ${\tilde{C}}$ depending on *s*, *N* and $M'$. This implies the continuity of $G_s$ with respect to the strong $L^2$-topology. This, along with the (well-known) lower semicontinuity of $G_1$ with respect the weak $H^1$-convergence yields the conclusion. $\square$

A crucial remark in our analysis is that $\mathscr {G}^1_s$ can be rewritten as a sum of two terms resembling the formulation (3.1) of the limit functional $\mathscr {G}^1$; one term continuously convergent (as $s\rightarrow 1^-$) and the other one monotonically increasing in *s*. This on the one hand will facilitate the proof of the $\Gamma$-convergence result, essentially reducing it to proving a pointwise limit, and on the other will allow us to transfer the coerciveness of $\mathscr {G}^1_{{\bar{s}}}$ (for some $\bar{s}$ fixed) to the equicoerciveness of the sequence $\mathscr {G}^1_s$.

##### Lemma 3

Let $u \in H_0^1(\Omega )$. Then for every $s \in (0,1)$, we have

3.13 $$\begin{aligned} \begin{aligned} \mathscr {G}^1_s(u)&= -\frac{N\omega _N}{s}\int _{\mathbb {R}^N} \vert u(x) \vert ^2 \, \,\textrm{d}x+ 2 \int _{\mathbb {R}^N}\int _{B(0,1)^c} \frac{u(x+h)u(x)}{\vert h \vert ^{N+2s}} \,\textrm{d}h\,\textrm{d}x \\&\qquad +\int _{\mathbb {R}^N} \int _{B(0,1)} \frac{\int _{0}^{1} \vert \nabla u(x+th) \cdot h \vert ^2 \,\textrm{d}t-\vert u(x+h)-u(x)\vert ^2}{\vert h \vert ^{N+2s}} \,\textrm{d}h \, \,\textrm{d}x. \end{aligned}\nonumber \\ \end{aligned}$$

##### Proof

We observe that for all $u \in H_0^1(\Omega )$

3.14 $$\begin{aligned}&\int _{\mathbb {R}^N} \int _{\mathbb {R}^N} \frac{\vert u(x+h)-u(x) \vert ^2}{\vert h \vert ^{N+2s}}\,\textrm{d}h\,\textrm{d}x \nonumber \\&\quad = \frac{N\omega _N}{s}\int _{\mathbb {R}^N} \vert u(x) \vert ^2 \,\textrm{d}x-2\int _{\mathbb {R}^N}\int _{B(0,1)^c} \frac{u(x+h)u(x)}{\vert h \vert ^{N+2s}} \,\textrm{d}h\,\textrm{d}x \nonumber \\&\quad \qquad + \int _{\mathbb {R}^N} \int _{B(0,1)} \frac{\vert u(x+h)-u(x) \vert ^2}{\vert h \vert ^{N+2s}}\,\textrm{d}h\,\textrm{d}x. \end{aligned}$$

By summing and subtracting $\int _0^1|\nabla u(x+th)\cdot h|^2\,\textrm{d}t$, we rewrite the last term on the right-hand side of (3.14) as

3.15 $$\begin{aligned} \begin{aligned}&\int _{\mathbb {R}^N} \int _{B(0,1)} \frac{\vert u(x+h)-u(x) \vert ^2}{\vert h \vert ^{N+2s}}\,\textrm{d}h \,\textrm{d}x \\&\quad = \int _{\mathbb {R}^N} \int _{B(0,1)} \int _{0}^1 \frac{\vert \nabla u(x+th) \cdot h \vert ^2}{\vert h \vert ^{N+2s}} \,\textrm{d}t\,\textrm{d}h \,\textrm{d}x \\&\quad \qquad + \int _{\mathbb {R}^N} \int _{B(0,1)} \frac{\vert u(x+h)-u(x) \vert ^2 -\int _{0}^{1} \vert \nabla u(x+th) \cdot h \vert ^2 \,\textrm{d}t}{\vert h \vert ^{N+2s}} \,\textrm{d}h\,\textrm{d}x. \end{aligned}\nonumber \\ \end{aligned}$$

We now compute the first integral on the right-hand side above using the integration in polar coordinate and the change of variable $\xi =x+th$, denoting $\nu = \frac{h}{\vert h \vert }$;

3.16 $$\begin{aligned}&\int _{\mathbb {R}^N} \int _{B(0,1)} \int _{0}^{1} \frac{\vert \nabla u(x+th) \cdot \frac{h}{\vert h \vert } \vert ^2 }{\vert h\vert ^{N+2s-2}} \,\textrm{d}t\,\textrm{d}h\,\textrm{d}x \nonumber \\&\qquad \qquad =\int _{\mathbb {R}^N}\int _{B(0,1)}\int _{0}^{1} \frac{\vert \nabla u (\xi ) \cdot \frac{h}{\vert h \vert } \vert ^2}{\vert h \vert ^{N+2s-2}} \,\textrm{d}t \,\textrm{d}h \,\textrm{d}\xi \nonumber \\&\qquad \qquad = \int _{\mathbb {R}^N} \int _{0}^{1} \rho ^{1-2s} \int _{\partial B(0,1)} \vert \nabla u(\xi ) \cdot \nu \vert ^2 \,\textrm{d}{\mathcal {H}}^{N-1}(\nu ) \,\textrm{d}\rho \,\textrm{d}\xi \nonumber \\&\qquad \qquad = \frac{\omega _N}{2(1-s)} \int _{\mathbb {R}^N} \vert \nabla u(\xi ) \vert ^2 \,\textrm{d}\xi , \end{aligned}$$

where in the last step we have used that

$$\begin{aligned} \begin{aligned}&\int _{\partial B(0,1)} \vert \nabla u(\xi ) \cdot \nu \vert ^2 \,\,\textrm{d}{\mathcal {H}}^{N-1}(\nu ) \\&\qquad = \int _{\partial B(0,1)} \vert \nabla u(\xi ) \vert ^{2} \bigg | \frac{\nabla u(\xi ) }{\vert \nabla u(\xi )\vert }\cdot \nu \bigg |^2 \,\textrm{d}{\mathcal {H}}^{N-1}(\nu ) \\&\qquad =\vert \nabla u(\xi ) \vert ^{2} \int _{\partial B(0,1)} \vert e_i \cdot \nu \vert ^2 \,\textrm{d}{\mathcal {H}}^{N-1}(\nu ) \\&\qquad = \frac{\vert \nabla u(\xi ) \vert ^2}{N} \sum _{i=1}^{N} \int _{\partial B(0,1)} \vert e_i \cdot \nu \vert ^2 \,\textrm{d}{\mathcal {H}}^{N-1}(\nu )= \vert \nabla u(\xi ) \vert ^2 \omega _N. \end{aligned} \end{aligned}$$

Inserting formulas (3.15) and (3.16) into (3.14) we obtain

$$\begin{aligned} \begin{aligned}&\int _{\mathbb {R}^N} \int _{\mathbb {R}^N} \frac{\vert u(x+h)-u(x) \vert ^2}{\vert h \vert ^{N+2s}}\,\textrm{d}h\,\textrm{d}x- \frac{\omega _N}{2(1-s)} \int _{\mathbb {R}^N} \vert \nabla u(x) \vert ^2 \,\textrm{d}x \\&\qquad =\frac{N\omega _N}{s}\int _{\mathbb {R}^N} \vert u(x) \vert ^2 \,\textrm{d}x- \int _{\mathbb {R}^N} \int _{B(0,1)^c} \frac{u(x+h)u(x)}{\vert h \vert ^{N+2s}} \,\textrm{d}h \,\textrm{d}x \\&\qquad \qquad +\int _{\mathbb {R}^N} \int _{B(0,1)} \frac{\vert u(x+h)-u(x) \vert ^2 -\int _{0}^{1} \vert \nabla u(x+th) \cdot h \vert ^2 \,\textrm{d}t}{\vert h \vert ^{N+2s}} \,\textrm{d}h\,\textrm{d}x \end{aligned} \end{aligned}$$

and the claim is proved. $\square$

As commented above, the first two terms on the right-hand side of (3.13) converge locally uniformly as $s\rightarrow 1^-$. We then focus on the third term. For the sake of brevity, we introduce the following notation;

3.17 $$\begin{aligned} {\mathcal {J}}_s(u):=\int _{\mathbb {R}^N} \int _{B(0,1)} \frac{\int _{0}^{1} \vert \nabla u(x+th) \cdot h \vert ^2 \,\textrm{d}t-\vert u(x+h)-u(x)\vert ^2}{\vert h \vert ^{N+2s}} \,\textrm{d}h \,\textrm{d}x.\nonumber \\ \end{aligned}$$

In the next lemma we prove the positivity and monotonicity of ${\mathcal {J}}_s$ which will be useful to prove the $\Gamma$-convergence, see [8, 21].

##### Lemma 4

For $s\in (0,1)$ let ${\mathcal {J}}_s$ be defined as in (3.17). Then, for every $u\in H^1_0(\Omega )$, ${\mathcal {J}}_s(u)\ge 0$ and the map $s \mapsto {\mathcal {J}}_s(u)$ is monotone increasing in *s*, *i.e.* ${\mathcal {J}}_{s_1}(u)\le {\mathcal {J}}_{s_2}(u)$ for $0<s_1\le s_2<1$.

##### Proof

The claim is a straightforward consequence of the Fundamental Theorem of Calculus, Jensen’s inequality and the fact that $s\mapsto |h|^{-N-2s}$ is increasing in *B*(0, 1). $\square$

We end this section by noticing that for all $s \in (0,1]$ the functionals

3.18 $$\begin{aligned} (u,v) \mapsto \int _{\mathbb {R}^N} \int _{B(0,1)^c} \frac{u(x+h)v(x)}{\vert h \vert ^{N+2s}}\,\,\textrm{d}h\,\textrm{d}x \end{aligned}$$

are continuous in $L^2(\Omega ) \times L^2(\Omega )$ since, by Hölder’s inequality,

3.19 $$\begin{aligned} \Big |\int _{\mathbb {R}^N} \int _{B(0,1)^c} \frac{u(x+h)v(x)}{\vert h \vert ^{N+2s}}\,\,\textrm{d}h\,\textrm{d}x \Big | \le \frac{N\omega _N}{2s} \Vert u \Vert _{L^2(\Omega )} \Vert v \Vert _{L^2(\Omega )}. \end{aligned}$$

We will exploit this property quite often in the sequel.

#### Proof of the strong limsup inequality

We now prove the pointwise convergence $\mathscr {G}^1_s$ which, in particular, yields the strong limsup inequality, claimed in Theorem 2(iii).

##### Proof of Theorem 2(iii)

We claim that for every $u \in L^2(\Omega )$

3.20 $$\begin{aligned} \lim _{s\rightarrow 1^-} \mathscr {G}^1_s(u)= \mathscr {G}^1(u). \end{aligned}$$

By Lemma 3 it is sufficient to study the pointwise limit of ${\mathcal {J}}_s$ since the first two terms on the right-hand side of (3.13) converge pointwise (actually locally uniformly) to the same expression with $s=1$, *e.g.* by Lebesgue’s dominated convergence theorem. Indeed, since for all *h*, $x \in \mathbb {R}^N$

$$ \int _{0}^{1} \vert \nabla u(x+th) \cdot h \vert ^2 \,\textrm{d}t-\vert u(h+x)-u(x) \vert ^2\ge 0, $$

and ${\mathcal {J}}_s$ is increasing in *s* by Lemma 4, by monotone convergence

3.21 $$\begin{aligned} \lim _{s\rightarrow 1^-} {\mathcal {J}}_s(u) = \int _{\mathbb {R}^N} \int _{B(0,1)} \frac{\int _{0}^{1} \vert \nabla u(th+x) \cdot h \vert ^2 \,\textrm{d}t-\vert u(h+x)-u(x) \vert ^2}{\vert h \vert ^{N+2}}\,\textrm{d}h\,\textrm{d}x. \nonumber \\ \end{aligned}$$

This (as explained at the beginning of the proof) implies the pointwise limit of $\mathscr {G}^1_s$ which yields the claim by taking $u_n\equiv u$ as a recovery sequence.

The pointwise limit of the monotone family ${\mathcal {J}}_s$ directly implies the $\Gamma$-convergence of $\mathscr {G}^1_s$ to $\mathscr {G}^1$; indeed, increasing sequences $\Gamma$-converge to their pointwise limit (if this is lower semicontinuous) [21] and by stability under continuous perturbations [21].

Since we will prove the stronger Mosco-convergence, we need to provide first a compactness result.

#### Proof of the compactness

The next proposition is devoted to the proof of the compactness property claimed in Theorem 2(i). To prove it, we follow the strategy exploited in the proof of [14, Proposition 2.6], combining the monotonicity of ${\mathcal {J}}_s$ (Lemma 4) and the coercivity of the energy as *s* fixed (Proposition 1).

##### Proposition 2

Let $s_n \rightarrow 1^-$ and let $\mathscr {G}^1_s$ be defined as in (2.3). Let $\{ u_n\}_{n \in \mathbb {N}} \subset L^2(\Omega )$ be such that

$$ \Vert u_n\Vert _{L^2(\Omega )} \le m \quad \text {and} \quad \mathscr {G}_s^1(u_n) \le M $$

for all $n \in \mathbb {N}$, for some $m>0$ and $M\in \mathbb {R}$ independent of *n*. Then $\Vert \nabla u_n\Vert _{L^2(\Omega )}$ $\le M'$ for every $n \in \mathbb {N}$, for some $M'>0$ depending on *M*, *m* and *N*, and the sequence $\{u_n\}_{n \in \mathbb {N}}$ converges in the weak $H^1$-topology, up to a subsequence, to a function $u \in \mathfrak {D}(\mathscr {G}^1)$.

##### Proof

Without loss of generality, we can assume that $s_n\nearrow 1$. From Proposition 1 we have that $u_n \in H_0^1(\Omega )$ for all $n \in \mathbb {N}$. By Lemma 4 we obtain

3.22 $$\begin{aligned} \begin{aligned} |M| \ge \mathscr {G}_{s_n}^1(u_n)&= {\mathcal {J}}_{s_n}(u_n)-\int _{\mathbb {R}^N} \int _{B(0,1)^c} \frac{\vert u_n(x+h)-u_n(x) \vert ^2}{\vert h \vert ^{N+2s_n}}\,\textrm{d}h\,\textrm{d}x \\&\ge {\mathcal {J}}_{s_n}(u_n)-2 m^2\frac{N\omega _N}{s_n} \end{aligned} \end{aligned}$$

where in the last inequality we have used that

$$\begin{aligned}  &   \int _{\mathbb {R}^N} \int _{B(0,1)^c} \frac{\vert u_n(x+h)-u_n(x) \vert ^2}{\vert h \vert ^{N+2 s_n}}\,\textrm{d}h\,\textrm{d}x \\  &   \qquad = -2\int _{\mathbb {R}^N} \int _{B(0,1)^c} \frac{u_n(x+h)u_n(x) }{\vert h \vert ^{N+2 s_n}} \,\textrm{d}h\,\textrm{d}x+ \frac{N \omega _N}{s_n} \int _{\mathbb {R}^N} \vert u_n(x) \vert ^2 \,\textrm{d}x \end{aligned}$$

and inequality (3.19). Fix now $\bar{n} \in \mathbb {N}$ such that $s_{\bar{n}}>\frac{1}{2}$. We claim that there exists $M'$, depending on *M*, *m*, *N* but independent of *n*, such that $\Vert \nabla u_n\Vert _{L^2(\Omega )} \le M'$ for every $n\in \mathbb {N}$. Indeed, for every $n\ge \bar{n}$, using the monotonicity of $s \mapsto {\mathcal {J}}_s(u_n)$, see Lemma 4, and by (3.22) we have that

3.23 $$\begin{aligned} \begin{aligned} |M| +2m^2\frac{N\omega _N}{s_n}&\ge {\mathcal {J}}_{s_n}(u_n) \ge {\mathcal {J}}_{s_{\bar{n}}}(u_n) \\&= \mathscr {G}_{s_{\bar{n}}}^1(u_n)-\int _{\mathbb {R}^N} \int _{B(0,1)^c} \frac{\vert u_n(x+h)-u_n(x)\vert ^2}{\vert h \vert ^{N+2s_{\bar{n}}}}\,\textrm{d}h\,\textrm{d}x \\&\ge \mathscr {G}_{s_{\bar{n}}}^1(u_n) - 2N\omega _Nm^2. \end{aligned} \end{aligned}$$

This implies in particular that $\mathscr {G}_{s_{\bar{n}}}^1(u_n) \le \vert M \vert +6m^2\frac{N\omega _N}{s_n}$, and we obtain the claim using Proposition 1. Moreover, by (3.23) and Fubini’s theorem we get

$$\begin{aligned} \liminf _{n\rightarrow +\infty } \int _{B(0,1)}\int _{\mathbb {R}^N}\frac{|\nabla u_n(x)\cdot h|^2-|u_n(x+h)-u_n(x)|^2}{|h|^{N+2s_{{\bar{n}}}}}\,\textrm{d}x \,\textrm{d}h \\ = \liminf _{n\rightarrow +\infty } {\mathcal {J}}_{s_{{\bar{n}}}}(u_n) < +\infty . \end{aligned}$$

By weak $H^1$-convergence of $u_n$ to *u* (thus strong in $L^2$) and the (sequentially) weak lower semicontinuity of the Sobolev seminorm we further infer that

$$ \int _{B(0,1)}\int _{\mathbb {R}^N}\frac{|\nabla u(x)\cdot h|^2-|u(x+h)-u(x)|^2}{|h|^{N+2s_{{\bar{n}}}}}\,\textrm{d}x \,\textrm{d}h < +\infty . $$

Since this holds for every ${\bar{n}}\in \mathbb {N}$, by (3.21) we conclude that $u \in \mathfrak {D}(\mathscr {G}^1)$ and the result is proved. $\square$

#### Proof of the weak liminf inequality

We now turn to the proof of the weak liminf inequality in Theorem 2(ii). Before proving the desired result, we show it for the family $\{{\mathcal {J}}_s\}_{s \in (0,1)}$.

##### Lemma 5

Let ${\mathcal {J}}_s$ be defined as in (3.17). For every $s_n \nearrow 1$ and for all $u_n \rightharpoonup u$ in the weak $L^2$-topology such that

3.24 $$\begin{aligned} {\mathcal {J}}_{s_n}(u_n) \le M, \end{aligned}$$

for some $M>0$ independent of *n*, there holds

3.25 $$\begin{aligned} \begin{aligned}&\liminf _{n \rightarrow +\infty } {\mathcal {J}}_{s_n}(u_n) \\&\qquad \ge \int _{\mathbb {R}^N} \int _{B(0,1)} \frac{\int _{0}^{1} \vert \nabla u(th+x) \cdot h \vert ^2 \,\textrm{d}t-\vert u(x+h)-u(x) \vert ^2}{\vert h \vert ^{N+2}} \,\textrm{d}h \,\textrm{d}x. \end{aligned} \end{aligned}$$

##### Proof

Fix $\bar{s} <1$. By the monotonicity property provided by Lemma 4, (3.24) and (3.19) we get

$$ \mathscr {G}_{{\bar{s}}}^1(u_n) \le C. $$

Thus Proposition 1 yields that $u_n \rightharpoonup u$ in the weak $H^1$-topology. Therefore we have

$$\begin{aligned} \begin{aligned}&\liminf _{n \rightarrow + \infty } {\mathcal {J}}_{s_n}(u_n) \\&\qquad \ge \liminf _{n \rightarrow +\infty } {\mathcal {J}}_{{\bar{s}}} (u_n) \\&\qquad \ge \liminf _{n \rightarrow + \infty } \mathscr {G}_{{\bar{s}}}^1(u_n)+\lim _{n \rightarrow + \infty } \int _{\mathbb {R}^{N}} \int _{B(0,1)^{c}} \frac{\vert u_n(x+h)-u_n(x) \vert ^2}{\vert h \vert ^{N+2\bar{s}}}\,\textrm{d}h\,\textrm{d}x\\&\qquad \ge \mathscr {G}_{{\bar{s}}}^1(u)+ \int _{\mathbb {R}^{N}} \int _{B(0,1)^{c}} \frac{\vert u(x+h)-u(x) \vert ^2}{\vert h \vert ^{N+2\bar{s}}}\,\textrm{d}h\,\textrm{d}x = {\mathcal {J}}_{{\bar{s}}}(u) \end{aligned} \end{aligned}$$

where we have used for the last inequality the lower semicontinuity proved in Proposition 1, and the continuity of the map

$$ u \mapsto \int _{\mathbb {R}^N} \int _{B(0,1)^c} \frac{\vert u(x+h)-u(x) \vert ^2}{\vert h \vert ^{N+2 \bar{s}}} \,\textrm{d}h\,\textrm{d}x $$

with respect to the strong $L^2$-topology. Eventually, taking the limit as $\bar{s} \rightarrow 1^-$, the result follows by the pointwise convergence of ${\mathcal {J}}_{\bar{s}}$ (3.21).

We have now everything in place to give the proof of Theorem 2(ii), which then conclude completely the proof of Theorem 2.

##### Proof of Theorem 2(ii)

Let $s_n \nearrow 1$ and let $u_n \rightharpoonup u$ in the weak $L^2$-topology. We will prove that

$$\begin{aligned} \liminf _{n \rightarrow + \infty } \mathscr {G}_{s_n}^1(u_n) \ge \mathscr {G}^1(u). \end{aligned}$$

We can assume that $\liminf _{n \rightarrow + \infty } \mathscr {G}_{s_n}^1(u_n) < +\infty$, otherwise there is nothing to prove. By Proposition 2 we have $u_n \rightarrow u$ in the weak topology of $H^1(\Omega )$. By definition of ${\mathcal {J}}_{s_n}$, see formula (3.17), we have

3.26 $$\begin{aligned} \begin{aligned} \liminf _{n \rightarrow + \infty } \mathscr {G}_{s_n}^1(u_n)&\ge \liminf _{n \rightarrow + \infty } {\mathcal {J}}_{s_n}(u_n) \\&\quad + \liminf _{n \rightarrow + \infty } -\int _{\mathbb {R}^{N}} \int _{B(0,1)^{c}} \frac{\vert u_n(x+h)-u_n(x) \vert ^2}{\vert h \vert ^{N+2s_n}}\,\textrm{d}h\,\textrm{d}x. \end{aligned} \end{aligned}$$

Since $u_n \rightarrow u$ in $L^2(\Omega )$, we can assume that $u_n\rightarrow u$ almost everywhere, up to a subsequence, and there exists $f \in L^2(\Omega )$ such that $\vert u_n \vert \le f$. Hence by Lebesgue’s dominated convergence theorem we have

$$\begin{aligned} \lim _{n\rightarrow +\infty } \int _{\mathbb {R}^{N}} \int _{B(0,1)^{c}} \frac{\vert u_n(x+h)-u_n(x) \vert ^2}{\vert h \vert ^{N+2s_n}}\,\textrm{d}h\,\textrm{d}x \\ = \int _{\mathbb {R}^{N}} \int _{B(0,1)^{c}} \frac{\vert u(x+h)-u(x) \vert ^2}{\vert h \vert ^{N+2}}\,\textrm{d}h\,\textrm{d}x. \end{aligned}$$

By Lemma 5, from (3.26) and the formula above we infer that

$$\begin{aligned} \liminf _{n \rightarrow + \infty } \mathscr {G}^1_{s_n}(u) \ge -\frac{2}{N \omega _N }\int _{\mathbb {R}^N} \vert u(x) \vert ^2 \, \,\textrm{d}x+2 \int _{\mathbb {R}^N} \int _{B(0,1)^c} \frac{u(x+h)u(x)}{\vert h \vert ^{N+2}} \,\textrm{d}h\,\textrm{d}x \\ + \int _{\mathbb {R}^N} \int _{B(0,1)}\frac{\int _{0}^{1} \vert \nabla u(x+th) \cdot h \vert ^2 \,\textrm{d}t-\vert u(x+h)-u(x) \vert ^2}{\vert h \vert ^{N+2}}\,\textrm{d}h \,\textrm{d}x. \end{aligned}$$

This is the desired result, recalling (3.1). $\square$

## Stability of $\mathscr {G}_s^1$ gradient flows under $\Gamma$-convergence

In this section we investigate the stability of the $\mathscr {G}_s^1$ gradient flows as $s\rightarrow 1^-$. After computing the first variation of $\mathscr {G}_s^1$ for all $s\in (0,1)$ and of $\mathscr {G}^1$, we will recall and apply a general result of convergence of gradient flows of $\Gamma$-converging functionals that are uniformly $\lambda$*-convex* and $\lambda$*-positive* (see [19, Definition 3.1]). We will then prove that $\{\mathscr {G}_s^1\}_{s \in (0,1)}$ complies with these two latter properties, and hence we will obtain the desired stability result.

Here and below $\langle \cdot ,\cdot \rangle$ denotes the standard scalar product in $L^2(\Omega )$. In this section we denote by $v_t$ the partial time derivative of a function *v*.

### First variation of $\mathscr {G}_s^1$ and $\mathscr {G}^1$

For the convenience of the reader, we recall the definition and the main properties of the *s*-fractional Laplacian in the lines below, as it is part of the first variation of our rate energy $\mathscr {G}^1_s$.

For every $\phi \in C^\infty _c(\mathbb {R}^N)$ we define the *s*-fractional Laplacian of $\phi$ as

$$\begin{aligned} (- \Delta )^{s}\phi (x):=C(N,s)\lim _{r \rightarrow 0^+} \int _{\mathbb {R}^N \setminus B(0,r)} \frac{\phi (x)-\phi (x+h)}{\vert h \vert ^{N+2s}} \,\textrm{d}h \end{aligned}$$

for every $x \in \mathbb {R}^N$, where *C*(*N*, *s*) is the multiplicative factor

$$ C(N,s):=\Big (\int _{\mathbb {R}^N}\frac{1-\cos (h_1)}{|h|^{N+2s}}\,\textrm{d}h\Big )^{-1}, $$

see *e.g.* [29, formulas (3.1) and (3.2)]. It is well-known (cf. [29, Corollary 4.2]) that *C*(*N*, *s*) behaves like $(1-s)$ as $s\rightarrow 1^{-}$, in the sense that $\frac{C(N,s)}{1-s}\rightarrow \frac{4}{\omega _N}$. In [29, Lemma 3.2] it is proved that for all $\phi \in C_c^{\infty }(\mathbb {R}^N)$, $(-\Delta )^s \phi \in L^{\infty }(\mathbb {R}^N)$ and

$$\begin{aligned} (-\Delta )^s \phi (x) = C(N,s)\frac{1}{2}\int _{\mathbb {R}^N} \frac{2 \phi (x) -\phi (x+h)-\phi (x-h)}{\vert h \vert ^{N+2s}} \,\textrm{d}h. \end{aligned}$$

It is well-known that the *s*-fractional Laplacian is related to the squared *s*-Gagliardo seminorm, *i.e.* the *s*-fractional laplacian is the first variation of the functional

$$u \mapsto \frac{C(N,s)}{4(1-s)}G_s(u)=\frac{C(N,s)}{4} \int _{\mathbb {R}^N} \int _{\mathbb {R}^N} \frac{ \vert u(y)-u(x)\vert ^2}{\vert y-x \vert ^{N+2s}}\,\textrm{d}y\,\textrm{d}x,$$

with $G_s$ defined as in (2.1), for a proof of this see [19, Proposiotion 4.1]. As customary, we extend the fractional Laplacian to every $u \in L^2(\Omega )$ such that $G_s(u)< \infty$ (namely $u\in H_0^s(\Omega )$) via duality, by defining the *s*-fractional Laplacian of *u* as follows

$$\begin{aligned} \langle (-\Delta )^su,\phi \rangle := \langle u,(-\Delta )^s\phi \rangle \quad \text {for all } \phi \in C^{\infty }_{c}(\Omega ). \end{aligned}$$

For all $u \in H_0^1(\Omega )$ and for all $\phi \in C_c^{\infty }(\Omega )$ we have that the first variation of $u \mapsto \mathscr {G}_s^1(u)$ is

4.1 $$\begin{aligned} \lim _{t \rightarrow 0} \frac{ \mathscr {G}_s^1(u+t \phi )- \mathscr {G}_s^1(u)}{t}=\Big \langle u,\frac{\omega _N(-\Delta )-\frac{4(1-s)}{C(N,s)}(-\Delta )^s}{1-s}\phi \Big \rangle . \end{aligned}$$

We now define a new operator that we will prove to be the first variation of the $\Gamma$-limit $\mathscr {G}^1$. Let $\phi \in C^\infty _c(\mathbb {R}^N)$ we define

4.2 $$\begin{aligned} \begin{aligned} \mathfrak {L} \phi (x):=&-2N \omega _N \phi (x)+ 4\int _{B(0,1)^c} \frac{\phi (x+h)}{\vert h \vert ^{N+2}}\,\textrm{d}h \\&\qquad -2\int _{B(0,1)} \frac{2\phi (x)-\phi (x-h)-\phi (x+h)+ h \cdot \nabla ^2 \phi (x)h }{\vert h \vert ^{N+2}} \,\textrm{d}h. \end{aligned} \end{aligned}$$

The operator $\mathfrak {L}$ defined in (4.2) is well-posed as results by Lemma 6 below.

#### Lemma 6

Let $$ \phi \in C_c^3(\Omega )$$. Then $$ \mathfrak {L} \phi \in C(\Omega )$$.

#### Proof

We first notice that

4.3 $$\begin{aligned} \sup _{x \in \Omega }\Big | \int _{B(0,1)^c} \frac{\phi (x+h)}{\vert h \vert ^{N+2}}\,\textrm{d}h\Big | \le \Vert \phi \Vert _{L^{\infty }} \int _{B(0,1)^c} \frac{1}{\vert h \vert ^{N+2}} \,\textrm{d}h = \frac{N \omega _N}{2} \Vert \phi \Vert _{L^{\infty }}. \end{aligned}$$

Using the Taylor expansion of the function $\phi$ we have that for all $(x,h) \in \Omega _1 \times B(0,1)$

4.4 $$\begin{aligned} \big \vert 2\phi (x)-\phi (x-h)-\phi (x+h)+ h \cdot \nabla ^2 \phi (x)h \big \vert \le R\vert h \vert ^3 \end{aligned}$$

where $$\Omega _1:= \{z \in \mathbb {R}^N\, : \, \textrm{dist}(z, \Omega ) \le 1\}$$ and

$$ R:= C(N)\sup \Big \{ \Big \vert \frac{\partial ^3 \phi }{\partial x_i \, \partial x_j \partial x_k}(x) \Big \vert \, : x \in \Omega _1, \, i,\,j,\,k = 1, \dots ,N \Big \}. $$

Therefore by formula (4.4) we obtain

4.5 $$\begin{aligned} \begin{aligned}&\sup _{x \in \Omega }\Big | \int _{B(0,1) } \frac{2\phi (x)-\phi (x-h)-\phi (x+h) + h \cdot \nabla ^2 \phi (x)h }{\vert h \vert ^{N+2}} \,\textrm{d}h \Big | \\&\hspace{35ex} \le R \int _{B(0,1)} \frac{1}{\vert h \vert ^{N-1}} \,\textrm{d}h = R N \omega _N. \end{aligned} \end{aligned}$$

By (4.3) and (4.5) we obtain $\mathfrak {L} \phi \in L^{\infty }(\Omega )$. The continuity of $x \mapsto \mathfrak {L} \phi (x)$ is a consequence of Lebesgue’s dominated convergence theorem, which can be applied by exploiting (4.5) and yields sequential continuity.

As for the *s*-fractional Laplacian, for every $u \in \mathfrak {D}(\mathscr {G}^1)$ we define $\mathfrak {L}u$ by duality as follows

$$\begin{aligned} \langle \mathfrak {L} u,\phi \rangle := \langle u,\mathfrak {L} \phi \rangle \quad \text {for all } \phi \in C^{\infty }_{c}(\Omega ). \end{aligned}$$

The operator $\mathfrak {L}$ is the first variation of the functional $\mathscr {G}^1$, as shown below.

#### Lemma 7

For every $u \in \mathfrak {D}(\mathscr {G}^1)$ and for every $\phi \in C_{c}^{\infty }(\Omega )$ we have

4.6 $$\begin{aligned} \lim _{t \rightarrow 0} \frac{ \mathscr {G}^1(u+t\phi )-\mathscr {G}^1(u)}{t}= \langle \mathfrak {L} u, \, \phi \rangle . \end{aligned}$$

#### Proof

To shorten the computations, we denote the functionals ${\mathcal {A}},{\mathcal {B}} :L^2(\Omega ) \rightarrow (-\infty ,+\infty )$ as

$$\begin{aligned} {\mathcal {A}}(u):= -N \omega _N \int _{\mathbb {R}^N} \vert u(x) \vert ^2 \, \,\textrm{d}x, \quad {\mathcal {B}}(u):= 2 \int _{\mathbb {R}^N}\int _{B(0,1)^c} \frac{u(x+h)u(x)}{\vert h \vert ^{N+2}}\,\textrm{d}h\,\textrm{d}x. \end{aligned}$$

For all $r \in (0,1)$ we also define the bilinear functionals ${\mathcal {C}}_r:L^2(\Omega )\times L^2(\Omega ) \rightarrow (-\infty ,+\infty ]$

$$\begin{aligned} {\mathcal {C}}_r(u,v):= &   - \int _{S_r}(u(h+x)-u(x)-\nabla u(x+th)\cdot h) \\  &   \,\times (v(h+x)-v(x)+\nabla v(x+th)\cdot h)\vert h \vert ^{-N-2} \,\textrm{d}x\,\textrm{d}h \,\textrm{d}t \end{aligned}$$

where $S_r:= \mathbb {R}^N \times \big (B(0,1) \setminus B(0,r)\big )\times (0,1)$. By (3.1), for all $u \in \mathfrak {D}(\mathscr {G}^1)$ we have that

$$\begin{aligned} \mathscr {G}^1(u)= {\mathcal {A}}(u)+{\mathcal {B}}(u)+ \lim _{r \rightarrow 0^+} {\mathcal {C}}_r(u,u). \end{aligned}$$

Hence, we have that

$$\begin{aligned} \lim _{t \rightarrow 0} \frac{ \mathscr {G}^1(u+t\phi )-\mathscr {G}^1(u)}{t} = \lim _{t \rightarrow 0} \frac{ {\mathcal {A}}(u+t\phi )-{\mathcal {A}}(u)}{t}+\lim _{t \rightarrow 0} \frac{ {\mathcal {B}}(u+t\phi )-{\mathcal {B}}(u)}{t}\\ +\lim _{t \rightarrow 0} \lim _{r \rightarrow 0^+}\frac{{\mathcal {C}}_r(u+t \phi , u+t \phi )-{\mathcal {C}}_r(u, u)}{t}. \end{aligned}$$

By standard algebraic computations, we obtain that

4.7 $$\begin{aligned} \begin{aligned} \lim _{t \rightarrow 0}&\frac{ {\mathcal {A}}(u+t\phi )-{\mathcal {A}}(u)}{t} +\lim _{t \rightarrow 0} \frac{ {\mathcal {B}}(u+t\phi )-{\mathcal {B}}(u)}{t} \\&= \int _{\mathbb {R}^N}u(x)\Big (-2N \omega _N\phi (x)+ 4\int _{B(0,1)^c} \frac{\phi (x+h) }{\vert h \vert ^{N+2}} \,\textrm{d}h\Big )\,\textrm{d}x. \end{aligned} \end{aligned}$$

From the bilinearity of the functional, we get that

4.8 $$\begin{aligned} {\mathcal {C}}_r(u+t \phi ,u+t \phi )= {\mathcal {C}}_r(u,u)+ t {\mathcal {C}}_r(u,\phi )+ t {\mathcal {C}}_r(\phi ,u)+ t^2 {\mathcal {C}}_r(\phi ,\phi ). \end{aligned}$$

Notice that, by (3.2) and Lemma 1, for all $r>0$ there holds $0\le {\mathcal {C}}_r(u,u)< +\infty$, $0\le {\mathcal {C}}_r(\phi ,\phi )< +\infty$ and $0\le {\mathcal {C}}_r(u+t \phi ,u+t \phi ) < +\infty$. Thus, by formula (4.8) above, we infer that ${\mathcal {C}}_r(u,\phi )+{\mathcal {C}}_r(\phi ,u)\ne \infty$ for all $r>0$. Therefore by (4.8) we obtain

$$\begin{aligned} \lim _{t \rightarrow 0} \lim _{r \rightarrow 0^+} \frac{{\mathcal {C}}_r(u+t \phi , u+t \phi )-{\mathcal {C}}_r(u, u)}{t}= \lim _{r \rightarrow 0^+}{\mathcal {C}}_r(u,\phi )+ {\mathcal {C}}_r(\phi ,u). \end{aligned}$$

We now claim that for every $r>0$

4.9 $$\begin{aligned} \begin{aligned}&{\mathcal {C}}_r(u,\phi )+ {\mathcal {C}}_r(\phi ,u)\\&= - 2 \int _{\mathbb {R}^N} u(x) \int _{B(0,1) \setminus B(0,r) } \hspace{-5ex} \frac{2\phi (x)-\phi (x-h)-\phi (x+h)+ h \cdot \nabla ^2 \phi (x)h }{\vert h \vert ^{N+2}} \,\textrm{d}h \,\textrm{d}x; \end{aligned} \end{aligned}$$

we prove (4.9) by expanding the product at the numerator of ${\mathcal {C}}_r(u,\phi )$ and ${\mathcal {C}}_r(\phi ,u)$ (cf. definition of ${\mathcal {C}}_r)$ and rewriting all the terms in a convenient way. Regarding the first term in *u* of ${\mathcal {C}}_r(u,\phi )$, by a change of variable we obtain that

4.10 $$\begin{aligned} \begin{aligned}&\int _{S_r}\frac{ u(h+x) (\phi (x+h)-\phi (x)+\nabla \phi (x+th)\cdot h)}{\vert h \vert ^{N+2}} \,\textrm{d}t\,\textrm{d}h \,\textrm{d}x \\&\qquad = \int _{S_r} u(x) \frac{\phi (x)-\phi (x-h)+\nabla \phi (x+(t-1)h)\cdot h}{\vert h \vert ^{N+2}} \,\textrm{d}t \,\textrm{d}h \,\textrm{d}x. \end{aligned} \end{aligned}$$

The second term in *u* simply reads

4.11 $$\begin{aligned} \int _{S_r}u(x)\frac{-\phi (x+h)+\phi (x)-\nabla \phi (x+th)\cdot h}{\vert h \vert ^{N+2}} \,\textrm{d}t\,\textrm{d}h \,\textrm{d}x. \end{aligned}$$

After an integration by parts and a change of variables, the third term reads

4.12 $$\begin{aligned} \begin{aligned}&\int _{S_r}\frac{-\nabla u(x+th)\cdot h (\phi (x+h)-\phi (x)+\nabla \phi (x+th)\cdot h)}{\vert h \vert ^{N+2}} \,\textrm{d}t\,\textrm{d}h \,\textrm{d}x \\&\quad =\int _{S_r} u(x+th)\frac{\nabla \phi (x+h)\cdot h- \nabla \phi (x)\cdot h+ h \cdot \nabla ^2 \phi (x+th) h}{\vert h \vert ^{N+2}} \,\textrm{d}t\,\textrm{d}h \,\textrm{d}x \\&\quad = \int _{S_r} u(x)\frac{\nabla \phi (x+(1-t)h)\cdot h- \nabla \phi (x -th)\cdot h+ h \cdot \nabla ^2 \phi (x) h}{\vert h \vert ^{N+2}} \,\textrm{d}t \,\textrm{d}h \,\textrm{d}x . \end{aligned}\nonumber \\ \end{aligned}$$

Hence, by (4.10), (4.11) and (4.12) we get

4.13 $$\begin{aligned} \begin{aligned}&{\mathcal {C}}_r(u,\phi ) = -\int _{S_r}u(x)\frac{2\phi (x)-\phi (x-h)-\phi (x+h)+h\cdot \nabla ^2\phi (x) h}{|h|^{N+2}}\,\textrm{d}t\,\textrm{d}h\,\textrm{d}x \\&-\int _{S_r} u(x)\frac{2\nabla \phi (x+(1-t)h)\cdot h-\nabla \phi (x+th)\cdot h-\nabla \phi (x-th)\cdot h}{|h|^{N+2}}\,\textrm{d}t\,\textrm{d}h\,\textrm{d}x. \end{aligned}\nonumber \\ \end{aligned}$$

Analogous calculations also give that

4.14 $$\begin{aligned} \begin{aligned}&{\mathcal {C}}_r(\phi ,u) = -\int _{S_r}u(x)\frac{2\phi (x)-\phi (x-h)-\phi (x+h)+h\cdot \nabla ^2\phi (x) h}{|h|^{N+2}}\,\textrm{d}t\,\textrm{d}h\,\textrm{d}x \\&+\int _{S_r}u(x)\frac{2\nabla \phi (x+(1-t)h)\cdot h-\nabla \phi (x+th)\cdot h-\nabla \phi (x-th)\cdot h}{|h|^{N+2}}\,\textrm{d}t\,\textrm{d}h\,\textrm{d}x. \end{aligned}\nonumber \\ \end{aligned}$$

Gathering (4.13) and (4.14) formula (4.9) is proved. Eventually, by (4.2), (4.7), (4.9) we have (4.6). $\square$

#### Fourier symbols of $\mathfrak {L}$

For completeness, we conclude the discussion about the new operator $\mathfrak {L}$ by computing its Fourier symbols. This comes as an immediate consequence of the next lemma in which we compute the Fourier transform of $\mathfrak {L} \phi$ for any $\phi \in C_c^{\infty }(\mathbb {R}^N)$.

##### Lemma 8

For every $\phi \in C_c^\infty (\mathbb {R}^N)$ we have that

$$\begin{aligned} {\mathcal {F}}[ \mathfrak {L} \phi ](\xi )= \bigg (-2 N \omega _N + 4 \int _{B(0,1)^c} \frac{\cos (\xi \cdot h) }{\vert h\vert ^{N+2}}dh +2m(\xi )\bigg ) \hat{\phi }(\xi ) \end{aligned}$$

where $\xi \mapsto m(\xi )$ is the function defined in (3.7).

##### Proof

The thesis follows directly by the definition of $\xi \mapsto m(\xi )$ and by the well known properties of the Fourier transform;

$$\begin{aligned} {\mathcal {F}}[\phi (\cdot +h)](\xi )= e^{-i \xi \cdot h} \hat{\phi }(\xi ), \quad {\mathcal {F}}[h \cdot \nabla ^2 \phi (\cdot ) h](\xi )= - \vert \xi \cdot h \vert ^2 \hat{\phi }(\xi ). \end{aligned}$$

Eventually, since $\rho \mapsto \sin (\xi \cdot (\rho \sigma ))$ is even for every $\xi \in \mathbb {R}^N$ and $\sigma \in \mathbb {S}^{N-1}$, the result is proved. $\square$

We notice that, an alternative proof of the lemma above may come from computing the first variation of the Fourier representation of $\mathscr {G}^1$ in $L^2(\mathbb {R}^N;\mathbb {C})$. Indeed, by the same arguments as in Section 3.2 we have

$$ \mathscr {G}^1(u)=\int _{\mathbb {R}^N} \Big (m(\xi ) -\int _{B(0,1)^c}\frac{2-2\cos (\xi \cdot h)}{|h|^{N+2}}\,\textrm{d}h\Big )|{\hat{u}}(\xi )|^2 \,\textrm{d}\xi , $$

for every $u\in \mathfrak {D}(\mathscr {G}^1)$. Straightforward computations lead to the same conclusion of Lemma 8.

##### Remark 2

From (3.10) and Lemma 8, we conclude that the symbols of the limit operator $\mathfrak {L}$ is comparable (*i.e.* coincides up to a scaling factor of order 1) to $|\xi |^2\log (|\xi |^2)$. Interestingly, this expression would appear when calculating the symbol of the formal derivative of $(-\Delta )^s$ as $s\rightarrow 1^-$, since

$$ \partial _s |\xi |^{2s}|_{s=1}=|\xi |^2\log (|\xi |^2). $$

Justified by the fact that the symbol of the logarithim Laplacian $\log (-\Delta )$ is indeed $\log (|\xi |^2)$ (see [16]), we are tempted to guess that $\mathfrak {L}$ relates to the operator $(-\Delta )\log (-\Delta )$, for instance in the sense that they are of the same “differentiability order”. It is not immediately clear whether the two operators coincide or not due to the different scaling factors. We however refrain from proceeding further in this direction with a rigorous analysis since this goes beyond the objective of this paper and we postpone it to future works.

### Abstract stability results for gradient flows

It is well known that, under suitable uniform convexity assumptions, $\Gamma$-convergence commutes with gradient flows, see [7, 11] and also [3, 42] for a generalization in metric spaces. For the specific framework of uniform $\lambda$-convex functionals we refer for instance to [19, 39, 40].

Here, we recall the stability result as appears in [19, Theorem 3.8], that best suits our purposes. We start by recalling the notion of $\lambda$-positive and $\lambda$-convex functions.

Let $\mathscr {H}$ be a Hilbert space endowed with the scalar product $\langle \cdot ,\cdot \rangle _\mathscr {H}$ and the norm $|\cdot |_\mathscr {H}$ and let $\lambda >0$. We say that a functional $\mathscr {F}: \mathscr {H}\rightarrow (-\infty ,+\infty ]$ is $\lambda$-convex if the functional $\mathscr {F}(\cdot )+ \frac{\lambda }{2} \vert \cdot \vert ^{2}_{\mathscr {H}}$ is convex. Moreover, we say that $\mathscr {F}$ is $\lambda$-positive if $\mathscr {F}(x)+ \frac{\lambda }{2} \vert x \vert ^{2}_{\mathscr {H}} \ge 0$ for every $x \in \mathscr {H}$.

In the sequel, we will write $\mathfrak {D}(\mathscr {F})= \{ x\in \mathscr {H}:\mathscr {F}(x)\ne + \infty \}$. For every $x\in \mathfrak {D}(\mathscr {F})$, $\partial \mathscr {F}(x)$ will denote the *subdifferential* of $\mathscr {F}$ at *x*, namely the set

$$ \partial \mathscr {F}(x) := \Big \{v\in \mathscr {H}:\liminf _{y\rightarrow x} \frac{\mathscr {F}(y)-\mathscr {F}(x)-\langle v,y-x\rangle _\mathscr {H}}{|y-x|_\mathscr {H}}\ge 0 \Big \}. $$

#### Theorem 3

Let $n\in \mathbb {N}$ and let $\mathscr {F}^n:\mathscr {H}\rightarrow (-\infty ,+\infty ]$ be a sequence of proper, strongly lower-semicontinuous functionals which are $\lambda$-convex and $\lambda$-positive, for some $\lambda >0$ independent of *n*. Let $x_0^n\in \mathfrak {D}(\mathscr {F}^n)$ be such that

$$ \sup _{n\in \mathbb {N}}\mathscr {F}^n(x_0^n)<+\infty , $$

and $x_0^n\rightarrow x^\infty _0\in \mathscr {H}$ for some $x^\infty _0$. Assume that one of the following statements is satisfied:

- The functionals $\mathscr {F}^n$ are equicoercive and Mosco-converge to some proper functional $\mathscr {F}^\infty$ with respect to the weak/strong $\mathscr {H}$-convergence;
- The functionals $\mathscr {F}^n$ $\Gamma$-converge to some proper functional $\mathscr {F}^\infty$ with respect to the strong $\mathscr {H}$-convergence and every sequence $\{y^n\}_{n\in \mathbb {N}}\subset \mathscr {H}$ with
  $$ \sup _{n\in \mathbb {N}}\Big \{\mathscr {F}^n(y^n)+\frac{\lambda }{2}|y^n|_\mathscr {H}^2\Big \}<+\infty , $$
  admits a strongly convergent subsequence.

Then, $x_0^\infty \in \mathfrak {D}(\mathscr {F}^{\infty })$ and every $n\in \mathbb {N}$, there exist unique solutions $x^n$ to the problem

$$\begin{aligned} {\left\{ \begin{array}{ll} {\dot{x}}(t)\in -\partial \mathscr {F}^n(x(t)) \qquad \text {for a.e.\ }{t\in (0,+\infty )}, \\ x(0)=x_0^n \end{array}\right. } \end{aligned}$$

and $x^\infty$ to the problem

$$\begin{aligned} {\left\{ \begin{array}{ll} {\dot{x}}(t)\in -\partial \mathscr {F}^\infty (x(t)) \qquad \text {for a.e.\ }{t\in (0,+\infty )}, \\ x(0)=x^\infty _0, \end{array}\right. } \end{aligned}$$

and $x^n$ weakly converge to $x^\infty$ in $H^1_{ loc}([0,+\infty );\mathscr {H})$.

Furthermore, if additionally

$$\begin{aligned} \lim _{n\rightarrow +\infty } \mathscr {F}^n(x^n_0)=\mathscr {F}^\infty (x^\infty _0)\,, \end{aligned}$$

then, $x^n$ strongly converge to $x^\infty$ in $H^1_{ loc}([0,+\infty );\mathscr {H})$, $x^n(t)\overset{\mathscr {H}}{\rightarrow }x^{\infty }(t)$ and $\mathscr {F}^n(x^n(t))\rightarrow \mathscr {F}^{\infty }(x^{\infty }(t))$ for every $t\ge 0$.

We now state the following general result which connects the notion of first variation with that of *subgradient* (*i.e.* an element of the subdifferential). For a proof of the result below we refer to [19, Proposition 3.7]. For every vector space $\mathscr {V}$ we denote by $\mathscr {V}^*$ the algebraic dual space of $\mathscr {V}$ and by $\mathscr {V}'$ the topological dual space of $\mathscr {V}$.

#### Proposition 3

Let $\mathscr {F}:\mathscr {H}\rightarrow (-\infty ,+\infty ]$ be a proper lower semicontinuous functional which is $\lambda$-convex, for some $\lambda >0$ and let $x\in \mathfrak {D}(\mathscr {F})$. Let ${\hat{\mathscr {H}}}$ be a dense subspace of $\mathscr {H}$. If there exists $T\in ({\hat{\mathscr {H}}})^*$ such that

$$\begin{aligned} \lim _{t\rightarrow 0}\frac{\mathscr {F}(x+ty)-\mathscr {F}(x)}{t}=T(y)\qquad \text {for every }y\in {\hat{\mathscr {H}}}\,, \end{aligned}$$

then, either $\partial \mathscr {F}(x)=\emptyset$ or $\partial \mathscr {F}(x)=\{v\}$, where *v* is the (unique) element in $\mathscr {H}$ satisfying $T(y)=\langle v,y\rangle _\mathscr {H}$ for every $y\in {\hat{\mathscr {H}}}$. In particular, $T\in ({\hat{\mathscr {H}}})'$ and *v* is its unique continuous extension to $\mathscr {H}'$.

### Convergence of the $\mathscr {G}_s^1$ gradient flows to the gradient flows of $\mathscr {G}^1$

Our strategy to prove the convergence of the gradient flows of the $\mathscr {G}_s^1$ energies is to prove that the family $\{\mathscr {G}_s^1\}_{s\in (\frac{1}{2},1)}$ satisfies the hypotheses of Theorem 3. To this end, we only need to prove the uniform $\lambda$-positivity and $\lambda$-convexity as the hypothesis that energy $\mathscr {G}_s^1$ converge in sense of Mosco to $\mathscr {G}^1$ is already proved in the first part of the article.

#### Lemma 9

For every $\lambda > 8 N\omega _N$, the functionals $\mathscr {G}_s^1$ are $\lambda$-positive and $\lambda$-convex for every $s \in \left( \frac{1}{2},1\right)$.

#### Proof

We start by noticing that, by decomposition (3.13) and by the positivity result provided by Lemma 4, in order to prove the $\lambda$-positivity of $\mathscr {G}^1_s$ it is sufficient to prove that

$$ \Big (\frac{\lambda }{2}-\frac{N\omega _N}{s}\Big )\int _{\mathbb {R}^N}|u(x)|^2 \,\textrm{d}x+ 2\int _{\mathbb {R}^N}\int _{B(0,1)^c}\frac{u(x+h)u(x)}{|h|^{N+2s}} \,\textrm{d}h \,\textrm{d}x\ge 0. $$

This, in turn, is an immediate consequence of (3.19) and the fact that $s\ge \frac{1}{2}$.

Now we show that the functionals $\mathscr {G}_s^1$ are $\lambda$-convex for every $s \in \left( \frac{1}{2},1\right)$. For every $u,v\in L^2(\Omega )$ we define the function $f:\mathbb {R}\rightarrow \mathbb {R}$ as

$$\begin{aligned} f(t):= &   \Big (\frac{\lambda }{2}-\frac{N\omega _N}{s}\Big )\int _{\mathbb {R}^N}|u(x)+t v(x)|^2 \,\textrm{d}x \\  &   + 2\int _{\mathbb {R}^N}\int _{B(0,1)^c}\frac{(u(x+h)+t v(x+h))(u(x)+t v(x))}{|h|^{N+2s}} \,\textrm{d}h \,\textrm{d}x \end{aligned}$$

and claim that $\frac{d^2}{d t^2}f(t) \ge 0$ for every $t \in \mathbb {R}$. Indeed, again by (3.19) we obtain

$$\begin{aligned} \frac{d^2}{d t^2}f(t)&= \Big (\lambda -\frac{2N\omega _N}{s}\Big )\int _{\mathbb {R}^N}|v(x)|^2\,\textrm{d}x+4\int _{\mathbb {R}^N}\int _{B(0,1)^c}\frac{v(x+h)v(x)}{|h|^{N+2s}} \,\textrm{d}h \,\textrm{d}x \\&\ge \big (\lambda -8N \omega _N\big ) \int _{\mathbb {R}^N}|v(x)|^2\,\textrm{d}x \ge 0. \end{aligned}$$

With analogous computations, defining $g:\mathbb {R}\rightarrow \mathbb {R}$ as $g(t):={\mathcal {J}}_s(u+tv)$ we obtain, as a consequence again of Lemma 4,

$$ \frac{d^2}{d t^2}g(t) = 2 {\mathcal {J}}_s(v,v)\ge 0. $$

Thanks to decomposition (3.13), the two inequalities above then yield the result. $\square$

We have now everything in place to prove the convergence of the gradient flows corresponding to the $\mathscr {G}_s^1$ functionals as $s \rightarrow 1^-$. The strategy of the proof follows [19, Theorems 4.4–4.6] and [27, Theorems 4.2–4.6].

#### Theorem 4

Let $\{s_n\}_{n\in \mathbb {N}}\subset (0,1)$ be such that $s_n\rightarrow 1^-$ as $n\rightarrow +\infty$. Let $u^\infty _0\in L^2(\Omega )$ and let $\{u^{n}_0\}_{n\in \mathbb {N}}\subset H_0^1(\Omega )$ be such that $\sup _{n\in \mathbb {N}}\mathscr {G}^1_{s_n}(u^n_0)<+\infty$ and $u^n_0\rightarrow u^\infty _0$ in $L^2(\Omega )$. Then, for every $n\in \mathbb {N}$ there exists a unique solution $u^n\in H^1_{loc}([0,+\infty ); L^2(\Omega ))\cap L^\infty ([0,+\infty );H^1_0(\Omega ))$ to

4.15 $$\begin{aligned} {\left\{ \begin{array}{ll} u_t(t) = -\frac{\omega _N(-\Delta )-\frac{4(1-s)}{C(N,s)}(-\Delta )^s}{1-s} u(t) \qquad \text {for a.e.\ }t\in (0,+\infty )\,, \\ u(0)=u^{n}_0\,, \end{array}\right. } \end{aligned}$$

such that $u^n(t)\in H^1_0(\Omega )$ for $a.e.\, t\ge 0$. Moreover, $u^\infty _0\in \mathfrak {D}(\mathscr {G}^1)$ and $u^n\rightarrow u^\infty$ in the weak topology of $H^1_{ loc}([0,+\infty );L^2(\Omega ))$ as $n\rightarrow +\infty$ , where $u^\infty \in H^1_{loc}([0,+\infty );L^2(\Omega ))\cap L^\infty ([0,+\infty );H^1_0(\Omega ))$ is the unique solution to

4.16 $$\begin{aligned} {\left\{ \begin{array}{ll} u_t(t) =-\mathfrak {L} u(t)\qquad \text {for a.e.\ }t\in (0,+\infty ) \\ u(0)=u^\infty _0\,, \end{array}\right. } \end{aligned}$$

and such that $u^\infty (t)\in \mathfrak {D}(\mathscr {G}^1)$ for $a.e. \,t\ge 0$ .

Furthermore, if additionally

$$ \lim _{n\rightarrow +\infty } \mathscr {G}_{s_n}^1(u^n_0)= \mathscr {G}^1(u^\infty _0)\,, $$

then, $u^n\rightarrow u^\infty$ strongly in $H^1_{ loc}([0,+\infty );L^2(\Omega ))$ and

$$ \Vert u^n(t)-u^\infty (t)\Vert _{L^2(\Omega )}\rightarrow 0,\quad \mathscr {G}_{s_n}^1(u^n(t))\rightarrow \mathscr {G}^1(u^\infty (t)),\quad \text {for every }t\ge 0\,. $$

#### Proof

By the very definition of $\mathscr {G}_s^1$, see (2.3), the domain of $\mathscr {G}_{s_n}^1$ is $H_0^1(\Omega )$ for all $n \in \mathbb {N}$ and from Proposition 1$\mathscr {G}^1_{s_n}$ is lower semicontinuous with respect to the strong $L^2$ topology. By Lemma 9$\mathscr {G}^1_{s_n}$ is $\lambda$-positive and $\lambda$-convex for every $\lambda > 4N \omega _N+4 \vert \Omega \vert$; here we can assume for simplicity and without loss of generality that $s_n\ge \frac{1}{2}$ for every $n\in \mathbb {N}$.

Now by formula (4.1) and Proposition 3 applied with ${\mathcal {F}}= \mathscr {G}^1_{s_n}$, $\mathscr {H}= L^2(\Omega )$ and $\hat{\mathscr {H}}= C_c^{\infty }(\Omega )$ we have that for every $u \in H^1_0(\Omega )$, either $\partial \mathscr {G}_{s_n}^1(u)= \emptyset$ or

$$ \partial \mathscr {G}_{s_n}^1(u)=\Big \{\frac{\omega _N(-\Delta )-\frac{4(1-s)}{C(N,s)}(-\Delta )^s}{1-s}u\Big \}, $$

with

$$ \frac{\omega _N(-\Delta )-\frac{4(1-s)}{C(N,s)}(-\Delta )^s}{1-s} u \in L^2(\Omega ). $$

Furthermore, by Lemma 7 and by Proposition 3 applied with ${\mathcal {F}}= \mathscr {G}^1$, $\mathscr {H}=L^2(\Omega )$ and $\hat{\mathscr {H}}= C_c^{\infty }(\Omega )$ we have that every $u \in \mathfrak {D}(\mathscr {G}^1)$, either $\partial \mathscr {G}^1(u)= \emptyset$ or $\partial \mathscr {G}^1(u)= \{\mathfrak {L} u\}$ with $\mathfrak {L} u \in L^2(\Omega )$.

By Theorem 3 the solutions to problems (4.15) and (4.16) exist and are unique and belong to $L^2(\Omega )$ for *a*.*e*. $t\ge 0$. The fact that $\sup _{t\in [0,+\infty )}\Vert u^n(t)\Vert _{H^1}$ $<+\infty$ is a direct consequence of Proposition 2 and the fact that

$$ \sup _n \mathscr {G}^1_{s_n}(u^n(t))\le \sup _n \mathscr {G}^1_{s_n}(u_0^n)<+\infty . $$

This together with Theorem 2 (i) yields also that $\sup _{t\in [0,+\infty )}\Vert u^\infty (t)\Vert _{H^1}<+\infty$. Eventually, the stability claim follows by applying Theorem 3 with $\mathscr {H}=L^2(\Omega )$, ${\mathcal {F}}_n= \mathscr {G}_{s_n}^1$ and ${\mathcal {F}}^{\infty }= \mathscr {G}^1$, once noticed that, in view of Theorem 2,both assumptions (*a*) and (*b*) of Theorem 3 are satisfied. $\square$
